# Automated Multi-Wavelength Quality Assessment of Photoplethysmography Signals Using Modulation Spectrum Shape Features

**DOI:** 10.3390/s23125606

**Published:** 2023-06-15

**Authors:** Abhishek Tiwari, Gordon Gray, Parker Bondi, Amin Mahnam, Tiago H. Falk

**Affiliations:** 1 Institut National de la Recherche Scientifique, University of Quebec, Montreal, QC H5A 1K6, Canada; abhishek.tiwari@myant.ca; 2 Myant Inc., Toronto, ON M9W 5Z9, Canada; gordon.gray@myant.ca (G.G.); parker.bondi@myant.ca (P.B.); amin.mahnam@myant.ca (A.M.)

**Keywords:** photoplethysmogram, modulation spectrogram, quality assessment, wearable devices

## Abstract

Photoplethysmography (PPG) is used to measure blood volume changes in the microvascular bed of tissue. Information about these changes along time can be used for estimation of various physiological parameters, such as heart rate variability, arterial stiffness, and blood pressure, to name a few. As a result, PPG has become a popular biological modality and is widely used in wearable health devices. However, accurate measurement of various physiological parameters requires good-quality PPG signals. Therefore, various signal quality indexes (SQIs) for PPG signals have been proposed. These metrics have usually been based on statistical, frequency, and/or template analyses. The modulation spectrogram representation, however, captures the second-order periodicities of a signal and has been shown to provide useful quality cues for electrocardiograms and speech signals. In this work, we propose a new PPG quality metric based on properties of the modulation spectrum. The proposed metric is tested using data collected from subjects while they performed various activity tasks contaminating the PPG signals. Experiments on this multi-wavelength PPG dataset show the combination of proposed and benchmark measures significantly outperforming several benchmark SQIs with improvements of 21.3% BACC (balanced accuracy) for green, 21.6% BACC for red, and 19.0% BACC for infrared wavelengths, respectively, for PPG quality detection tasks. The proposed metrics also generalize for cross-wavelength PPG quality detection tasks.

## 1. Introduction

The photoplethysmogram (PPG) has become an ubiquitous physiological modality in clinical and consumer devices due to its noninvasive nature and cost-effectiveness [1,2]. PPG makes use of transmission, absorption, and reflection of optical radiation in peripheral tissue to measure changes in blood volume levels in the microvascular bed of well-perfused tissues, such as the surface of the fingertip, the wrist, the earlobe, and the forehead [1,3]. Traditionally, PPG has been used for the measurement of heart rate and blood oxygen saturation levels at rest. However, the recent boom in the use of wearable devices for wellbeing [4] and telehealth monitoring [5,6] applications has drawn attention to other health and physiological information present in the PPG signal.

It is known, for example, that the PPG signal is influenced by various sources, such as heart (including heart rate, heart rate variability, and stroke volume), circulation system (including cardiovascular properties such as arterial stiffness and blood pressure), and other physiological processes, including respiration and the autonomic nervous system [1]. As such, recent research has focused on, e.g., continuous cuffless blood pressure measurement [7], arterial stiffness detection [8], arrhythmia detection [9], and sleep staging [10] from the PPG signal, just to name a few applications. These applications typically rely on morphological analysis of the PPG signal, which in turn require high-quality signals to detect certain characteristics, such as diastolic and systolic peaks, or the dicrotic notch.

PPG signal quality and morphology, however, is sensitive to various different sources, including ambient light, applied pressure at the measurement site, measurement site location, skin tone, and motion [11]. Sensor movement due to motion, in particular, remains one of the main sources of noise which can severely impact the quality of the recorded signal [11,12]. Motion-induced noise is often periodic, with frequency components corresponding to the movement being performed (e.g., walking, running), often degrading the signal and affecting subsequent analyses [12,13]. As a result, methods to estimate and remove this noise have often relied on the use of a reference accelerometer signal [12,13,14,15]. Signal quality assessment for PPG signals during motion without the use of an accelerometer is still an underexplored domain, but could provide great benefits for health-monitoring applications with limited-resource, low-cost devices.

Signal quality assessment is usually performed via the extraction of various signal quality indexes (SQIs) followed by a heuristic rule-based system or a pattern recognition method to distinguish between good- and bad-quality data. To date, signal metrics have been extracted using statistical-, temporal-, frequency-, and template-based approaches [1]. Time- and frequency-based metrics are the most widely reported in the literature. In [16], for example, eight SQIs were tested, including SQIs based on the skewness, kurtosis, and zero-crossing rate of the PPG signal, as well as one based on the frequency content of the signal. Skewness was shown to outperform all other metrics. In [17], zero-crossing rate and signal-autocorrelation-based SQIs were combined with a threshold-based rule-set to assess PPG signal quality. The work in [18], in turn, extracted 71 different SQIs (including statistical-, frequency-, and template-based ones) and used feature selection to pick the top-performing ones, i.e., number of zero-crossings, autocorrelation peaks, median noise ratio, and relative power per pulse. Lastly, the authors in [19] compared features proposed in [16,18] and showed that the first and second autocorrelation peaks, as well as PPG skewness, were the top-performing metrics.

Template-based SQIs require the segmentation of each beat by first calculation of pulse peak and derivation of individual beat templates. Then, a reference template is used to compute different SQIs, such as the Euclidean distance [20], correlation [21] or dynamic time warping distance [22], of individual beat template from the reference template. Individual SQIs on each beat template can also be calculated and averaged, as performed in [18]. These measures require the availability of a reference template and can be highly dependent on the accuracy of PPG peak-detection algorithm, which can be affected by the signal quality itself.

More recently, approaches based on machine learning have emerged. These can rely on conventional methods where features are first extracted and then applied to a classifier (e.g., [23]) or on end-to-end deep learning paradigms where decisions are made based on raw or transformed PPG signal inputs (e.g., [24,25]). The work in [24], for example, proposed the use of the short-time Fourier transformed version of the PPG signal as input to a convolutional neural network (CNN). The authors in [25], in turn, decomposed the PPG signal into various frequency components and generated a 2D image to serve as input to a lightweight CNN for signal quality assessment.

In this work, we deviate from these existing solutions and propose to use the so-called modulation spectrogram. This is a 2D representation of a time series which has shown usefulness across numerous applications based on wearable devices (e.g., [26,27]). The modulation spectrogram is able to separate the rates at which various signal components are modulated and differentiate them from noise. This property has been used for signal quality assessment [28] and enhancement [29] of electrocardiogram (ECG) signals. In this work, we propose a new modulation-spectrum-based quality metric for PPG signals and compare its performance to a number of benchmark methods.

The remainder of this paper is organized as follows: Section 2 describes the modulation spectrogram representation used in this paper along with the proposed SQI. Section 3 presents the experimental setup, where the device, data collection, preprocessing, feature extraction, classification pipelines, and figures of merit used are described. Section 4 presents the results obtained and discusses the top features. Finally, Section 5 presents the conclusions.

## 2. Modulation Spectrogram Representation and Proposed SQI

The spectrogram has become a popular tool for the signal processing of nonstationary signals. By providing information about the change of frequency components over time, it quantifies the short-term frequency fluctuations appearing in the signal [26]. However, commonly occurring noises often overlap with the signal of interest in the frequency domain, thus making this representation suboptimal for quality assessment. The modulation spectrum, in turn, is known to capture higher-order periodicities of the signal not otherwise obvious in the time and time–frequency domains. Additionally, by capturing the rate of change of frequency components, the modulation representation of the signal also isolates biologically-modulated components of the signal from randomly-varying noise, thus making it a good candidate for signal quality assessment. For biological signals, this property has been explored previously for affect recognition [30] and Alzheimer’s disease detection [31] using electroencephalography (EEG) signals, to quality assessment [28], enhancement [29], and noise-robust heart rate variability estimation [32] using ECGs.

### 2.1. Signal Processing Steps

**Figure 1:**
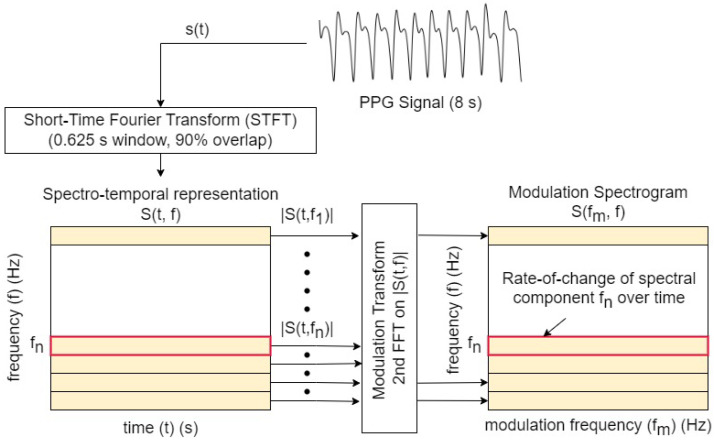
Signal-processing steps involved in the computation of the modulation spectrogram. Here, $f_{n}$ represents the nth frequency component in the spectrotemporal representation of the signal.

The signal processing steps involved in the computation of the modulation spectrogram are depicted in Figure 1. First, the signal s(t) is transformed to the time–frequency domain (spectrogram) via the short-time Fourier transform (STFT) (here, implemented using a 0.625 s moving window with 90% overlap). The choice of window size was made empirically based on initial experiments on clean PPGs.

The spectrotemporal representation S(t,f) can be written as the convolution of the time-domain signal s(t) and a series of complex filterbanks $\lambda_{f}\left(t\right)e^{j2\pi ft}$:

(1) $$S\left(t,f\right)=s\left(t\right)★\lambda_{f}\left(t\right)e^{j2\pi ft},$$

where convolution is indicated by ★, $e^{j2\pi ft}$ denotes complex oscillations for different frequencies *f*, and $\lambda_{f}\left(t\right)$ is the window applied for each complex oscillation at a given frequency. A second transform is then applied across the time axis, for each frequency bin magnitude |S(t,f)|. This results in a frequency–frequency representation of the signal termed ‘modulation spectrogram’ ($S(f_{m},f)$), which characterizes the rate of change of different spectral components. The modulation spectrogram associated with the signal *s* can be written as

(2) $$S\left(f_{m},f\right)=F_{t}\left\{|S(t,f)|\right\},$$

where $F_{t}\left\{\cdot \right\}$ indicates the the Fourier transform over the time dimension.

**Figure 2:**
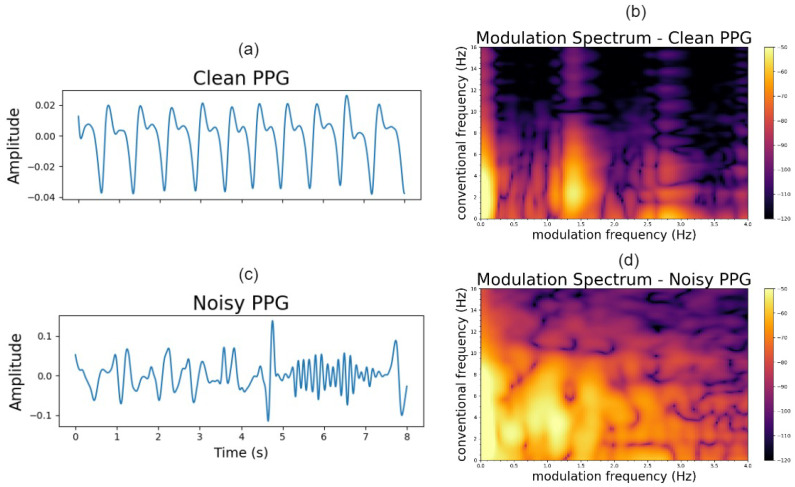
Clean (**a**) and noisy (**c**) PPG signals; modulation spectrogram of clean (**b**) and noisy (**d**) PPG.

Here, *f* is used to characterize the conventional frequency (Hz), and $f_{m}$ the modulation frequency. Clean and noisy PPG signals with their corresponding modulation spectrograms are shown in Figure 2. As can be seen, for the clean PPG, the signal is modulated by the heart rate causing the signal information being concentrated as lobes in the modulation domain. The modulation frequency of the first lobe corresponds to the heart rate of the signal, with other lobes appearing at harmonics of this fundamental frequency. This is to be expected, as a similar lobe-like structure appears in ECG signals which are modulated by similar biological processes [28]. In contrast, for a noisy PPG signal, the lobe information is not visible and is polluted by modulation components generated by noise. With these insights, we propose a quality metric that takes into account the spectral shape of the modulation lobes.

While one may choose to use the modulation spectrogram directly as input to a machine learning algorithm (i.e., to be treated as an image, similar to many applications relying on spectrograms [24,25]) for PPG quality assessment, this increases the model complexity and makes the system less interpretable. To improve the interpretability and simplicity of the proposed method, here, we decided to extract metrics from the modulation spectrogram and then apply them to machine learning algorithms.

### 2.2. Proposed SQI

**Figure 3:**
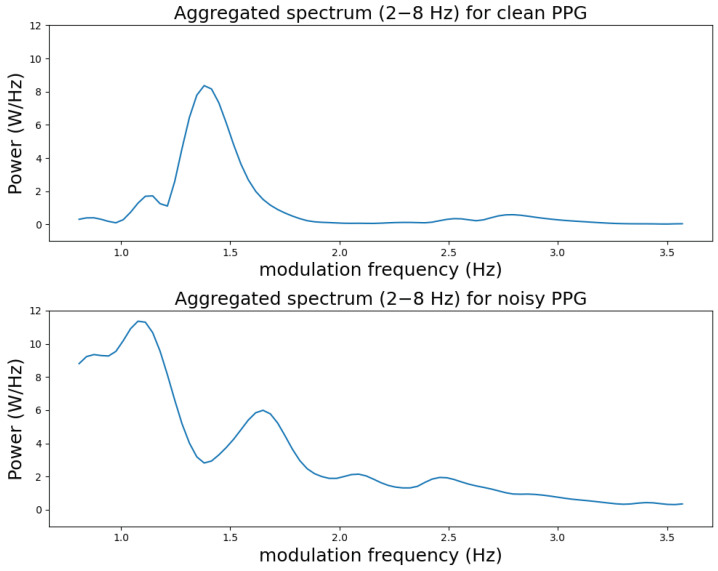
Aggregated spectra for clean and noisy PPG samples between 0–3.6 Hz modulation frequency. The aggregation was performed between the 2–8 Hz conventional frequency range.

The proposed SQIs are motivated by the fact that the spectral shape of the aggregated modulation spectrogram across conventional frequency is very different for clean and noisy PPGs. Figure 3 shows the modulation spectrum aggregated over conventional frequencies between 2 and 8 Hz from the modulation spectrograms in Figure 2b,d, respectively. We can see that the clean aggregated spectrum has one clear spectral peak while the noisy spectrum has multiple peaks in comparison.

**Figure 4:**
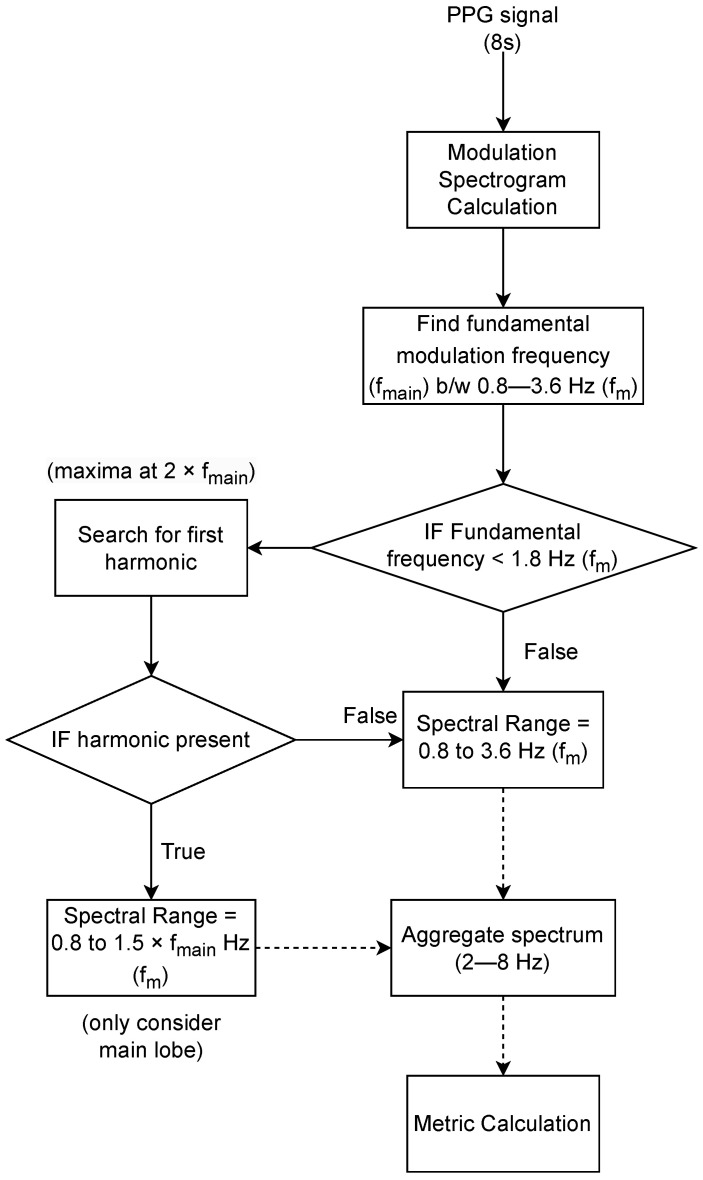
Steps for calculation of proposed SQI metrics. Here, ($f_{m}$) corresponds to modulation frequency in Hz.

The processing steps to compute the proposed SQI measures are summarized in the diagram shown in Figure 4. First, the modulation spectrogram is calculated over the 8 s signal window. Following this, the modulation frequency corresponding to the fundamental lobe ($f_{main}$) is calculated by locating the modulation frequency corresponding to the maximum of the aggregated spectrum along the conventional frequency axis between 2 to 8 Hz. The range 2 to 8 Hz was chosen for aggregation as most of the signal information lies within this region [16]. The $f_{main}$ corresponds to heart rate for a clean signal but can be arbitrary for noisy signals. If no harmonics of the signal exist in the considered range of modulation frequencies, the full spectral range between 0.8–3.6 Hz (48–216 bpm) can be used for aggregation. Otherwise, we look for the first harmonic, and in case it exists, we use a smaller spectral range between 0.8 and $1.5*f_{main}$ Hz so that only the main lobe is considered for calculation. A harmonic will only exist if the $f_{main}$ is greater than half the maximum modulation frequency considered (i.e., 3.6 Hz, 216 bpm) and has a harmonic spectral peak at $2*f_{main}$. Finally, three spectral descriptors of the aggregated spectrum are calculated in the specified range.

The three spectral descriptors include spectral entropy (entMS), spread (sprdMS), and crest (crstMS), defined as follows:

1. Spectral entropy:
  (3) $$entMS=\frac{-\sum_{k=b_{1}}^{b_{2}}p_{k}\log\left(p_{k}\right)}{\log(b_{2}-b_{1})},$$
  where $b_{1}$ and $b_{2}$ represent the range of the relevant frequency bands, and $p_{k}$ represents the spectral power for a given frequency bin *k*. Spectral entropy is the measure of uniformity of the spectrum.
2. Spread, or the standard deviation around the spectral centroid, is given by
  (4) $$sprdMS=\sqrt{\frac{\sum_{k=b_{1}}^{b_{2}}{\left(f_{k}-centroid\right)}^{2}p_{k}}{\sum_{k=b_{1}}^{b_{2}}p_{k}}},$$
  where centriod represents the centroid of the spectrum, calculated as follows:
  (5) $$centroid=\frac{\sum_{k=b_{1}}^{b_{2}}f_{k}.p_{k}}{\sum_{k=b_{1}}^{b_{2}}p_{k}},$$
  where $b_{1}$ and $b_{2}$ represent the range of the relevant frequency bands. $f_{k}$ represents the frequency in Hz for bin *k*, and $p_{k}$ represents the spectral power for that bin. The spread represents the instantaneous bandwidth of the spectrum.
3. Crest, which measures the ratio of the maximum of the spectrum to the mean of the spectrum, i.e.,
  (6) $$crstMS=\frac{max(p_{k\epsilon (b_{1}:b_{2})})}{\frac{1}{b_{2}-b_{1}}\sum_{k=b_{1}}^{b_{2}}p_{k}},$$
  where $b_{1}$ and $b_{2}$ represent the range of the relevant frequency bands, and $p_{k}$ represents the spectral power for a given frequency bin *k*. Crest is a measure of the peakedness of the spectrum.

All these metrics are focused on characterizing if the aggregated spectrum is contaminated closer to a uniform or single peak distribution. If the PPG signal is clean, the aggregated spectrum should have only the fundamental frequency peak, thus decreasing the spectral entropy, spread, and increasing the crest value. However, if motion-related noise exists, the signal contains other spectral components, making the spectrum more uniform in nature, resulting in the opposite of the effects mentioned above.

## 3. Experimental Setup

### 3.1. Device Description

**Figure 5:**
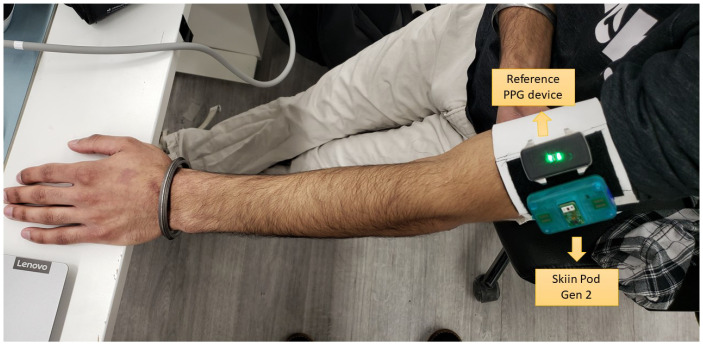
View of the Skiin Pod Gen2 placed on the arm along with the reference device.

A prototype of the Skiin Gen2 system (Myant Inc., Toronto, ON, Canada) was used to obtain accurate PPG signals. The device consists of a dedicated PPG analog front-end with auto-DC and ambient cancellation, along with other sensors that were not used in this experiment. An integrated optoelectronic sensor SFH7072 (OSRAM) was used for recording PPG which consisted of one red (peak λ = 660 nm), two green (526 nm), and one infrared (IR, 950 nm) LED sources, one large IR-cut photodetector (PD), and one small broadband PD. The PD–LED distances were 5.67 mm to broadband, 2.95 mm to IR-cut, and 5.67 mm to broadband for red, green, and IR LEDs, respectively. The prototype mechanical casing was 3D-printed with a window for the optoelectronic package to protrude above the case surface to directly touch skin and reduce any air gap. The total thickness of the device was 15 mm. The LED current was set uniquely for each subject, trial, and LED colour such that the DC level of the output PPG was the same across each (half-full-scale range). The sampling rate was set to 250 Hz. The device setup is shown in Figure 5.

### 3.2. Data Collection

Data used for the analysis were collected as part of a PPG measurement protocol. Various tests were conducted to change the blood pressure levels, while cuff-based measurement was performed intermittently. These tests consisted of several physical activity tasks, including performing squats for 1 min and walking on a treadmill for 4.5 min starting at 1 m/h with increments of 1 m/h per 45 s, in addition to staying stationary in different postures.

Data using this protocol were collected from eight participants. A balance between male and female participants was maintained and participants with different skin colour tones and varying blood pressure (BP) levels were invited to participate, as these are factors known to impact PPG quality [11]. Experiments were performed in line with the Helsinki declaration and the participants consented to participating. The study protocol was described to the participants in detail and they consented to participate and were allowed to cease participation anytime without providing any reason. The Skiin pod was used to measure continuous green, red, and infrared PPG from the arm. A reference continuous PPG monitor was placed next to the Skiin Pod (shown in Figure 5). The positions of the reference device and Skiin Pod were counterbalanced for each subject. Only the PPG data collected using the Skiin pod during the protocol were used herein.

### 3.3. Signal Annotation and Processing

As we are interested in motion-based noise, the breathing noise in the signal was attenuated using the Hilbert-based filtering described in [33]. The PPG data were then annotated using an in-house graphical user interface. The annotations were performed on three levels:

1. Good, when a signal is completely clean and the diastolic and systolic peaks and dicrotic notch are clearly visible.
2. Okay, when the periodicity of the signal is still visible (heart rate calculation possible); however, the abovementioned signal characteristics are no longer visible.
3. Noisy, signal characteristics not visible at all.

The annotators could select any okay or noisy quality regions appearing in the signal; all other regions were assumed to be clean. Representative okay and noisy segments, as labelled by the annotators, are shown in Figure 2a,c, respectively.

Following this, the signals were epoched into 8 s windows with a 4 s overlap. For each 8 s window, the final label was generated by calculating the percentage of signals labelled as clean, okay, and noisy, and the label with the highest percentage was chosen as the ground truth. The final annotated database is summarized in Table 1. For our analysis, only segments with a majority label of clean or noisy are used.

### 3.4. Benchmark SQIs

After epoching the signal, apart from the proposed modulation-spectrogram-based SQIs described earlier, several benchmark SQIs were extracted for comparison, including:

1. Zero-crossings (zcSQI): This is defined as the number of times the signal crosses the zero-line. A higher value corresponds to a noisier signal. This method was first proposed in [16] and also explored in [18,34].
2. Skewness (sSQI): A clean PPG signal has a normal distribution. Changes to that distribution because of noise can be reflected in changes to the skewness value. The metric was used in [34,35].
3. Kurtosis (kSQI): Similar to skewness, increased outliers in the signal change the kurtosis of the signal distribution. Kurtosis for PPG signal quality assessment was used in [34,35,36,37].
4. snrElgendi: This metric was proposed in [16] and it is defined as the variance of the absolute value of the signal divided by the variance of the signal. This method was also used in [18].
5. Autocorrelation peaks ($AC_{Peak1}$ and $AC_{Peak2}$): Clean PPG signals are quasi-periodic and strongly self-correlated. Therefore, characterizing this using the first and second autocorrelation peaks of the signal was proposed as metrics for quality assessment in [36] and also explored in [18].

Henceforth, these benchmark SQIs will be termed ‘bench${}_{stats}$’.

Moreover, for frequency-based SQIs, the power spectral density (Px) is first calculated using Welch’s method. Five frequency-based SQIs are then calculated as ratios of power in different regions. These include:

1. Relative power (RelP): This metric was used in [16,34]. It is defined as the ratio of Px(1–2.25 Hz) to Px(1–8 Hz), where Px(A–B Hz) represents power in frequencies between A to B Hz.
2. In-to-Out SQI (iorSQI): Ratio of power between Px(1–8 Hz) to Px(0–1 Hz and 8-fs/2 Hz).
3. fSNR: Ratio of Px(1–2.25 Hz) to Px(0–fs/2 Hz), where fs is the sampling frequency.

The fSNR and iorSQI were also calculated after ignoring the DC low-frequency component of the signal, resulting in two new SQIs:

1. iorSQI-no-DC: Ratio of Px(1–8 Hz) to Px(1–fs/2 Hz);
2. fSNR-no-DC: IRatio of Px(1–2.25 Hz) to Px(1–fs/2 Hz).

Henceforth, these benchmark SQIs will be termed ‘bench${}_{freq}$’.

Lastly, template-based SQIs are calculated by first calculating PPG-peaks for each epoch. The PPG-peaks were calculated using the multiple moving average (MMA) detector proposed in [33]. Peaks are detected using four different moving average windows of sizes 0.5, 1, 1.5, and 2 s. The final detected PPG peak is the intersection of the peaks detected with the moving windows. Following this, each beat template is extracted. For a given signal epoch, the size of each template around the detected PPG-peak is changed adaptively based on the median peak-to-peak interval value. Finally, five template based SQIs are extracted:

1. Median relative power (Med-RelP): For each beat template, the Welch-based power spectral density is first calculated. Following this, the ratio of Px(1–2.25 Hz) to Px(0–8 Hz) is computed. The final metric is the median value of this ratio across all templates. This metric was proposed in [18].
2. snrElgendi-per-beat (beat-Elg): The snrElgendi metric is calculated for each template and the median value is used. This was proposed in [18].
3. Skewness-per-beat (beat-sSQI): Skewness of each beat is calculated by considering each template as a probability distribution. The average value is used. The metric is motivated from the idea that a clean PPG signal looks similar to a left skewed distribution.
4. Kurtosis-per-beat (beat-kSQI): Similar to skewness-per-beat, the kurtosis value is calculated and averaged.
5. Cardio-SQI: Proposed in [33], this metric quantifies how many times a signal has a zero gradient (maxima or minima) compared the number of peaks detected in the signal using a peak-detector algorithm. As noise increases, the number of zero-gradients in the signal increases more than the number of peaks detected.

Henceforth, these benchmark SQIs will be termed ‘bench${}_{template}$’.

### 3.5. Classification and Figures of Merit

Binary classification was performed between clean and noisy epochs, with the various quality metrics used as input features for each of the PPG wavelengths. For evaluation, two different settings were used. First, a leave-P-subjects-out validation setup was used separately for each wavelength (green, red, and infrared), where the training and test splits were performed based on the subject ID such that both sets contained unique subjects. The value of P was set to 3, leading to a total of ${}^{8}C_{3}$ = 56 iterations, with 3 subjects in the test and 5 subjects in the train set for each iteration. Classification results reported are the average and standard deviation over the 56 runs. To assess metrics importance, we used feature selection to select the top three features at each iteration and looked at the top three most frequently occurring metrics in the top feature set over the 56 iterations, along with their average classifier weights. Additionally, the performance of quality metrics across the different wavelengths was evaluated. To perform this, each wavelength was used as the training data and the performance was evaluated on other two wavelengths. The top three features for each of the wavelengths were observed.

A logistic regression classifier was used as our machine learning model. Linear models are simple to interpret and can provide information about feature importance. Ideally, we want a quality metric to be able to linearly separate signals of different quality. Such separation can allow for a simple threshold-based heuristic system for quality assessment. As such, the classification results reported here are to be considered as a lower bound on possible achievable performance with more complex models (e.g., deep neural networks). Balanced accuracy (BACC), F1-score (F1), sensitivity (Sens), and specificity (Spec) were used as figures of merit. BACC is known to be robust to class imbalances, compared to accuracy’ it is defined as the average of class-wise accuracy (recall) of both positive and negative classes, and is less sensitive to data imbalance [38]. The metrics can be calculated as follows:

(7) $$BACC=\frac{Sens+Spec}{2},$$

where

(8) $$Sens=\frac{TP}{TP+FN},$$

(9) $$Spec=\frac{TN}{TN+FP},$$

(10) $$F1=\frac{2*TP}{2*TP+FP+FN}.$$

The parameter TP corresponds to true positives, FP to false positives, TN to true negatives, and FN to false negatives.

To assess feature importance, the minimum-redundancy maximum-relevance (mRMR) [39] feature selection method was used. This method optimizes two criteria simultaneously using mutual information. First, the maximum-relevance step maximizes the average mutual information between each feature and the input labels, while the minimum-redundancy step minimizes the mutual information between different features. The algorithm finds near-optimal features using forward selection, with the chosen features maximizing the combined max–min criteria. This feature selection method is used to select the top three features (equal to the number of proposed modulation SQIs). The implementation of the classifier and feature selection algorithms was carried out using the scikit learn toolbox [40] and pyMRMR [39] library, respectively.

## 4. Results and Discussion

In this section, we present the results of the different analyses performed. First, the area under the receiver operating characteristic curve (AUC-ROC) analysis of the different metrics is conducted for all wavelengths. Next, the classification results under two different evaluation strategies are presented.

### 4.1. AUC-ROC Analysis

For each SQI, the distribution of values for clean and noisy labelled epochs were obtained. From these two distributions, area under curve (AUC) [31,41] was computed as a metric of separability between the groups. The AUC-ROC value ranges between 0 to 1, with very high or very low values both indicating high separability using the SQI, and values close to 0.5 indicating poor separability. In order to perform comparison across the SQIs, any metric with a value of ≤0.5 is transformed as $AUC_{trans}=\left(1-AUC\right)$. The top five metrics for each wavelength according to $AUC_{trans}$ are shown in Table 2.

As can be seen, the proposed entMS measured ranked top across all wavelengths. Parameter beat-Elg, in turn, appeared as the second or third, thus corroborating findings from [18]. The measure entMS showed the highest separability for all three wavelengths with improvements of 5.22%, 6.25%, and 6.83% over the second best benchmark SQI for green, red, and infrared PPGs, respectively. The measure crtMS appeared to be one of the top SQIs for the red and infrared PPG signals.

### 4.2. Classification—Leave 3 Subjects Out

In this section, we describe the results obtained with the top-three benchmark proposed, and a fusion of SQIs in a classification setting using leave-three-subjects-out for each of the wavelengths. Table 3, Table 4 and Table 5 show the performance results for green, red, and infrared wavelengths, respectively. The first three rows present the results achieved when using the top-three ranked SQIs under the statistical benchmarks, frequency benchmarks, and template benchmarks. Rows 5 and 6, in turn, show the results achieved when these benchmark measures were fused. Lastly, rows 4 and 7 show the results achieved with the proposed measures, as well as when the measures were fused with all the benchmark ones (‘All’), respectively.

For all three wavelengths, individually, the proposed modulation SQIs outperformed not only all the other benchmark SQIs individually, but also their fused sets. For example, compared with the fused ‘bench${}_{stat,freq,template}$’ set, the proposed measures showed improvements of 23.5% in BACC and 2.6% in F1 for green, 18.9% in BACC and 9.11% in F1 for red, and 19.8% in BACC and 10.2% in F1 for infrared, respectively. Combining the proposed modulation features with the benchmark features leads to further improvements, outperforming the individual proposed feature set by 6.7% in BACC and 1.6% in F1 for green, 2.3% in BACC and 1.1% in F1 for red, and 3% in BACC and 1.8% in F1 for infrared, respectively, thus suggesting some complementary information between the different modalities. Pairwise significance testing (p<0.01) with Bonferroni correction was performed between the ‘All’ condition and all other conditions for each wavelength and showed significance in all cases.

Next, we explore the top-three selected SQIs across all the 56 iterations for the ‘All’ feature set. To this end, features were ranked based on their frequency of occurrence as the top feature ($freq_{top}$) after feature selection. Table 6 shows the top-three most frequently occurring SQIs along with their frequency of occurrence ($freq_{top}$) and average weight when used in the logistic regression classifier for each of the three wavelengths.

As can be seen, across the three wavelengths, entMS is always the most frequently occurring top feature, even appearing as a top feature set for all iterations for red and infrared PPG ($freq_{top}=100\%$). The average weight of the feature is also highest compared to other top features, showing that it is the most important feature for classification. Apart from entMS, zcSQI and elgSQI are also in the top feature set for at least two of the wavelengths. Zero-crossing has been explored widely in the literature [16,17,18] and was among the top SQI metrics when compared against 71 others in [18]. Measure elgSQI was first proposed in [16] and its median beat-wise alternate was also shown to be among the top performing metrics in [18]. While this SQI did not perform well in [18], here, it is among the top benchmark metrics. Skewness (sSQI), despite being a widely used metric (e.g., [16,17,18,23]), only appeared as a top metric in our experiment for green PPG signals.

These results corroborate those reported in Table 2, where entMS was shown to be the metric with the greatest separability between noisy and clean PPG for all wavelengths, followed by elgSQI. Interestingly, while zcSQI appeared as a top measure for classification for green and infrared wavelengths, it did not show up as an individual measure with high separability in Table 2. This is likely due to the fact that the measure is related to other features in the combined feature set; thus, the mRMR selection algorithm considered it redundant and removed it from the feature pool. In fact, zero-crossing measures provide a simple estimate of the energy content of the signal; thus, such details could have been encoded already with the bench${}_{freq}$ set.

### 4.3. Cross-Wavelength Classification

For this experiment, the data from a given wavelength were used to train the classifier; data collected from the other two wavelengths were then used as the unseen test set. As different wavelengths of the PPG signal have different sensitivities to noise and movement artifacts, cross-wavelength tests provide details on the generalizability of the measures for PPG quality assessment. Table 7 reports the cross-wavelength balanced accuracy results. Features selected for each wavelength correspond to those listed in Table 6.

The results suggest that training on infrared PPG results in the highest accuracy across the other wavelengths, thus making it the best candidate for generalizable PPQ quality assessment. In turn, training on green wavelength PPG resulted in the lowest cross-wavelength results. As green PPGs are known to be more robust to artifacts, an unseen test set potentially corrupted with artifacts could be the cause for the lower results observed. Overall, based on its ability to separate the clean and noisy PPG classes, its importance in noisy PPG detection, and cross-wavelength experiments, the proposed measure based on the spectral entropy of the aggregated modulation spectrum (entMS) was shown to be the best for PPG quality assessment.

### 4.4. Study Limitations

There are some limitations to our study. First, the main source of noise in the study was elicited by walking and running on the treadmill. While movement is the most common noise source for PPG signals in real-life conditions, other sources of noise, such as arm movements simulating day-to-day activities, changes to sensor tightness, or loss of PPG signals, were not investigated. Such activities may also allow for a three-label classification, including differentiating between clean, acceptable, and noisy/unacceptable PPG segments. Second, the epoch size of 8 s was used for analysis as it is considered optimal for modulation spectrogram extraction [28]. However, different features may be more sensitive to noise over different window sizes. Smaller window sizes for time- and frequency-domain features can be investigated in the future. Smaller window size also allows for closer-to-real-time feedback on signal quality.

## 5. Conclusions

In this work, we presented three new modulation-spectrogram-based metrics for quality assessment of PPG during an experimental protocol involving movement and activity. The proposed metrics were compared against several benchmarks widely used in the PPG SQI literature. Through various experiments, we showed that the spectral entropy of the aggregated modulation spectrum appears to be the most important metric to distinguish clean from noisy PPG and allows not only for accurate per-wavelength quality assessment, but also cross-wavelength.

## Figures and Tables

**Table 1:** Number of epochs for each quality label and PPG wavelength after annotation and epoching.

Quality	Green	Red	Infrared
Clean	5373	4749	4622
Okay	265	274	290
Noisy	627	1242	1353
Total	6265	6265	6265

**Table 2:** Top five (highest to lowest) SQIs with AUC for different PPG wavelengths (* are ranked based on AUC${}_{trans}$ value).

Green		Red		Infrared	
SQI	AUC	SQI	AUC	SQI	AUC
entMS	0.947	entMS	0.969	entMS	0.953
beat-Elg	0.900	beat-Elg	0.912	crstMS	0.092 *
cardioSQI	0.889	crstMS	0.093 *	beat-Elg	0.892
elgSQI	0.881	fSNR-no-DC	0.101 *	elgSQI	0.865
sSQI	0.875	elgSQI	0.893	fSNR-no-DC	0.139 *

**Table 3:** Performance comparison of clean vs. noisy PPG detection using different feature sets for green wavelength PPG (mean ± standard deviation over leave-3-subjects-out iterations presented for each metric).

Features	BACC	F1	Sens	Spec
bench${}_{stat}$	0.663 ± 0.066	0.896 ± 0.025	0.354 ± 0.127	0.972 ± 0.017
bench${}_{freq}$	0.622 ± 0.046	0.890 ± 0.020	0.256 ± 0.098	0.987 ± 0.098
bench${}_{template}$	0.594 ± 0.058	0.880 ± 0.026	0.200 ± 0.120	0.988 ± 0.012
**proposed**	**0.729** ± **0.037**	**0.915** ± **0.012**	**0.486** ± **0.085**	**0.972** ± **0.085**
bench${}_{stat,freq}$	0.676 ± 0.048	0.899 ± 0.025	0.381 ± 0.091	0.971 ± 0.091
bench${}_{stat,freq,template}$	0.641 ± 0.047	0.892 ± 0.022	0.305 ± 0.096	0.978 ± 0.096
All	0.778 ± 0.065	0.930 ± 0.017	0.580 ± 0.136	0.977 ± 0.136

**Table 4:** Performance comparison of clean vs. noisy PPG detection using different feature sets for red wavelength PPG (mean ± standard deviation over leave-3-subjects-out iterations presented for each metric).

Features	BACC	F1	Sens	Spec
bench${}_{stat}$	0.749 ± 0.057	0.847 ± 0.033	0.569 ± 0.132	0.928 ± 0.034
bench${}_{freq}$	0.630 ± 0.057	0.797 ± 0.057	0.276 ± 0.125	0.984 ± 0.016
bench${}_{template}$	0.761 ± 0.068	0.858 ± 0.037	0.577 ± 0.151	0.945 ± 0.025
**proposed**	**0.884** ± **0.019**	**0.922** ± **0.015**	**0.820** ± **0.046**	**0.948** ± **0.019**
bench${}_{stat,freq}$	0.740 ± 0.057	0.846 ± 0.034	0.543 ± 0.134	0.936 ± 0.033
bench${}_{stat,freq,template}$	0.743 ± 0.067	0.845 ± 0.043	0.550 ± 0.151	0.936 ± 0.037
All	0.904 ± 0.015	0.932 ± 0.014	0.859 ± 0.042	0.950 ± 0.022

**Table 5:** Performance comparison of clean vs. noisy PPG detection using different feature sets for infrared wavelength PPG (mean ± standard deviation over leave-3-subjects-out iterations presented for each metric).

Features	BACC	F1	Sens	Spec
bench${}_{stat}$	0.709 ± 0.038	0.812 ± 0.031	0.498 ± 0.115	0.919 ± 0.062
bench${}_{freq}$	0.615 ± 0.045	0.767 ± 0.058	0.256 ± 0.106	0.974 ± 0.023
bench${}_{template}$	0.733 ± 0.061	0.835 ± 0.033	0.529 ± 0.133	0.937 ± 0.021
**proposed**	**0.857** ± **0.021**	**0.898** ± **0.021**	**0.784** ± **0.049**	**0.931** ± **0.028**
bench${}_{stat,freq}$	0.704 ± 0.043	0.81 ± 0.04	0.484 ± 0.129	0.925 ± 0.063
bench${}_{stat,freq,template}$	0.715 ± 0.061	0.815 ± 0.048	0.507 ± 0.149	0.924 ± 0.062
All	0.883 ± 0.024	0.914 ± 0.020	0.828 ± 0.052	0.938 ± 0.026

**Table 6:** Most important top features for each of the wavelengths.

Green			Red			Infrared		
Feature	${\mathrm{freq}}_{\mathrm{top}}$	Weight	Feature	${\mathrm{freq}}_{\mathrm{top}}$	Weight	Feature	${\mathrm{freq}}_{\mathrm{top}}$	Weight
entMS	91.1	7.965	entMS	100.0	10.220	entMS	100.0	9.490
sSQI	82.1	3.883	beat-kSQI	75.0	1.972	elgSQI	64.3	4.617
zcSQI	35.7	4.899	elgSQI	51.8	4.076	zcSQI	62.5	4.837

**Table 7:** Cross-wavelength BACC comparison.

Training/Testing	Green	Red	Infrared
Green	-	0.824	0.808
Red	0.870	-	0.895
Infrared	0.886	0.911	-

## Data Availability

Data is unavailable due to privacy or ethical restrictions.
